# Targeting RAGE-signaling pathways in the repair of rotator-cuff injury

**DOI:** 10.1007/s11010-024-05132-8

**Published:** 2024-10-12

**Authors:** Vikrant Rai, Vinitha Deepu, Devendra K. Agrawal

**Affiliations:** Department of Translational Research, Western University of Health Sciences, 309 E. Second Street, Pomona, CA 91766-1854 USA

**Keywords:** Chronic inflammation, Protein kinase C epsilon, RAGE, Rotator cuff, Signaling pathways

## Abstract

Rotator cuff injury (RCI) is a common musculoskeletal problem that can have a significant impact on the quality of life and functional abilities of those affected. Novel therapies, including proteomics-based, stem cells, platelet-rich plasma, and exosomes, are being developed to promote rotator-cuff healing. The receptor for advanced glycation end-products (RAGE) is a multifunctional receptor that is expressed on several cell types and is implicated in several physiologic and pathological processes, such as tissue repair, inflammation, and degeneration. Because of its capacity to bind with a variety of ligands and initiate signaling pathways that lead to inflammatory responses in RCI, RAGE plays a crucial role in inflammation. In this critical review article, we discussed the role of RAGE-mediated persistent inflammation in RCI followed by novel factors including PKCs, TIRAP, DIAPH1, and factors related to muscle injury with their therapeutic potential in RCI. These factors involve various aspects of muscle injury and signaling and the possibility of targeting these factors to improve the clinical outcomes in RCI still needs further investigation.

## Introduction

The rotator cuff is a group of muscles and associated tendons (supraspinatus, infraspinatus, teres minor, and subscapularis) in the shoulder that enable a broad range of motion while preserving the stability of the glenohumeral joint. The shoulder joint relies on the stability and mobility provided by the rotator cuff. Rotator cuff injury (RCI) is a common musculoskeletal problem that can have a significant impact on the quality of life and functional abilities of those affected [1]. Every year, millions of people worldwide suffer from RCI, particularly athletes and the elderly due to the tendon’s high-retear rate and slow-healing process. Patients’ motor abilities are severely compromised by this illness, which also lowers their quality of life. RCI repair is frequently influenced by the patient’s age, the size of the tear, and additional conditions like diabetes, hypercholesterolemia, obesity, and smoking [2]. The formation of scar tissue after RCI repair is another issue because the newly formed structure remains unable to fully restore the original biomechanical properties, which can easily lead to an increase in the retear rate. In addition to alternative treatments, arthroscopic and open surgery play a significant role in accelerating the healing process of rotator-cuff tears. In clinical practice, several kinds of biologic stimuli have been used. Proteomics-based, stem cells, platelet-rich plasma [3], and exosomes [4] are the most used biologics in laboratory and clinical research.

The role of hypercholesterolemia, oxidative stress, aging, and inflammation along with the detailed underlying mechanism, three models of tendon health, role of bone health in RCI, and mitochondrial dysfunction have been elaborately discussed and reported by us [2, 5]. Further, the role of alteration in the expression of collagen (COL)I and COLIII and its effects on extracellular matrix (ECM) remodeling and rotator-cuff tendon pathology have been reported [6], An upregulation of high-mobility group box protein (HMGB)-1 and NOD-, LRR- and pyrin domain-containing protein (NLRP) 3 inflammasomes activation in the injured rotator-cuff tendon is associated with chronic inflammation and ECM disorganization [7]. Further, an interaction of triggering receptor expressed on myeloid cells (TREM)-1 with HMGB-1 and receptor for advanced glycation end products (RAGE) contributes to chronicity of inflammation resulting in glenohumeral arthritis and rotator-cuff degeneration [8]. An interaction of TREM-1, AMP-activated protein kinase (AMPK), and microRNAs also plays a role in rotator-cuff pathology [9]. After a rotator-cuff tear, multiple factors play a role in the healing and fibrosis of the injured tissue, and abnormal accumulation of these cells affects the healing process. These cells include tenocytes, macrophages, myofibroblasts, and scleraxis-lineage cells [10]. These findings suggest that rotator-cuff injury as well as healing after injury is a multifactorial process and multifaceted aspects are needed for successful repair.

Our previous publication suggests that repair to the injured tendon causes an increased amount of fibrous tissue, water content, and fatty infiltration to the repair site probably contributing to the decreased mechanical properties of the injured tendons leading to an adaptation to chronic load [11]. This decrease in the biomechanical properties of the tendon is associated with hyperlipidemia [12]. However, it should be noted that hyperlipidemia may induce pathological changes in the tendon but without any changes in biomechanical properties [13]. This suggests that repair contributes to alteration in biomechanical properties. Further, increased activity of mitochondrial activity is associated with healing response and mitochondrial biogenesis may be a therapeutic target [5, 14]. We have discussed the existing treatments and the factors influencing the repair after surgery [2]. The hybrid-interpenetrating hydrogel network favors the bidirectional migration of tenocytes for rotator-cuff tendon regeneration [15], despite the presence of current-treatment strategies, emerging research with preclinical studies, failure of repair the rotator-cuff tendon due to comorbidities and recurrence of RCI warrant investigation of better therapeutic targets.

One area of increasing interest in the study of RCI is the receptor for advanced glycation end-products (RAGE) because of the involvement of inflammation in RCI pathogenesis. RAGE is a versatile receptor expressed on different cell types and is involved in a variety of physiologic and pathologic processes, including inflammation, tissue degeneration, and repair. Numerous signaling events linked to diabetes and its complications [2, 16], neurologic conditions [17], inflammation [18], and acute injuries such as RCI involve RAGE signaling. RAGE plays a pivotal role in inflammation due to its ability to interact with various ligands, triggering-signaling pathways contributing to inflammatory responses in RCI [19]. The complexity of RAGE involvement in inflammation arises due to its highly specific cytoplasmic domain that is involved in intracellular signaling upon ligand binding [18, 20, 21]. Inflammation is accelerated by a positive feed loop mechanism through the binding of RAGE to other effector molecules and an increase of cytokine secretion from immune cells at the site of injury [22]. Its complex nature and potential relevance to RCI make it an intriguing subject of investigation.

RAGE has been discussed as an attractive target in shoulder joint pathologies including RCI [2, 5, 8, 19, 21], however, no definitive therapy has been designed to target RAGE-ligand-mediated activation of inflammatory signaling. This may be due to the complex nature and isoforms of RAGE. Accumulation of AGEs in rotator-cuff tendons [23, 24] suggests RAGE as an attractive target in RCI. Further, the association of increased levels of the soluble form of RAGE (sRAGE), which functions as an antagonist of RAGE and reduces inflammation, with the severity of inflammatory diseases and inflammatory conditions like arthritis supports the notion of targeting RAGE in RCI. Due to the nonavailability of definitive treatment for RCI, it is beneficial to develop targeted interventions and improve the efficacy of current-treatment strategies to comprehend the role of RAGE in the pathogenesis of these injuries.

As discussed above, RCI has a multifactorial pathology, and we need to target multiple factors. RAGE is a novel target of interest in RCI to attenuate inflammation and promote ECM regeneration and thus promoting RCI healing. However, there is no definitive treatment strategy to target RAGE in RCI. This review briefly discusses the structure of RAGE, its activation, and the role in RCI followed by the possibility of targeting various factors involved in RAGE signaling. We have also discussed the aspects of RAGE activation after muscle injury due to the secretion of various factors related to muscle injury and the role of altered RAGE regulation via activated Protein Kinase C Epsilon (PKC-ξ) signaling. To our knowledge, there is no literature discussing the role of and targeting muscle-related factors and PKC-ξ in RCI repair to consider them as therapeutic targets in addition to conventional inflammatory targets and COL modulation.

## Pathogenesis of RCI and RAGE activation

### Tissue regeneration post-RCI and Factors affecting the RCI chronicity

An injury to the rotator-cuff triggers the body’s healing process. The blood vessels expand during the initial phase of inflammation, and immune cells infiltrate the injury site [25]. These immune cells eliminate damaged tissue and start the healing process, which starts a proliferative phase, in conjunction with other factors. In a damaged or torn tendon fiber, proliferating endothelial cells initiate the formation of new vessels and activated fibroblasts help in laying the ECM providing a bed for neo-angiogenesis, COL reorganization, and mediating repair of the damaged tissue [26]. The injured area regains strength and function because the newly formed tissue matures and reorganizes during the remodeling phase which includes reorientation and strengthening of the repaired tissue.

The rotator cuff’s natural-repair mechanism may not work in pathological conditions such as diabetes, hyperlipidemia, and obesity [2] or during repetitive injuries [27] and results in impaired repair. If the rate of production of reactive oxygen species (ROS) exceeds the antioxidant capacity of the tendon, it can trigger an inflammatory response and cause damage to the tendon by activating nuclear factor kappa beta (NF-kB) pathway or ROS-activated protein kinase B (AKT)/ forkhead box protein O1 (FOXO1) pathway [28]. It led to tendon fibrosis, adhesion, and scarring after an acute tendon injury [2] followed by restricted blood supply to the rotator-cuff tendons [28]. Poor vascularity makes it difficult for the immune cells and nutrients to be delivered, which is essential for effective healing. Age-related degeneration or persistent wear and tear can also weaken the tendon over time, reducing its ability to begin a successful repair response. The rotator cuff can become damaged cumulatively and unable to heal properly due to persistent strain or recurrent injuries. Research has also confirmed that peritendinous adhesions resulting from oxidative stress can also exacerbate inflammation by affecting factors such as tumor necrosis factor-alpha (TNF-α), transforming growth factor beta (TGF-β), COL1, superoxide dismutase (SOD)1, SOD2, and hypoxia-inducible factor (HIF)-1 α [29].

Increased blood lipid levels, such as high triglycerides or cholesterol (hyperlipidemia/hypercholesterolemia), have been linked to decreased blood flow and poor tendon healing, which may have an impact on the rotator cuff’s capacity to heal. The entrapment of low-density lipoprotein (LDL) particles in the dense regular COL of rotator-cuff tendons due to hyperlipidemia causes foam cell aggregation and macrophage recruitment. Persistent inflammation and the breakdown of the ECM in the rotator cuff were caused by subsequent inflammatory pathways, such as the Janus kinase 2/signal transducer and activator of transcription 3 (JAK2/STAT3) pathway and the nucleotide-binding domain, leucine-rich repeat containing protein 3 (NLRP3) inflammasome pathway [21, 30].

Being overweight or obese can put excessive strain on the rotator-cuff tendons, which may result in overuse over time, degeneration, and a reduced ability to heal from mechanical stress. Microtrauma in the rotator-cuff tendons due to repetitive injury can also result in cumulative damage and block the healing process. These elements have the potential to interfere with the natural healing cascade, which could result in decreased tissue regeneration, more degeneration, and a risk of getting rotator-cuff injuries worse. Increased risk of retear and persistent inflammation are two ways that hyperlipidemia restricts arthroscopic surgery. To maximize healing and restore shoulder function, management strategies for RCI frequently include addressing these contributing factors in addition to focused rehabilitation and, in certain cases, surgical intervention [31].

**Fig. 1:**
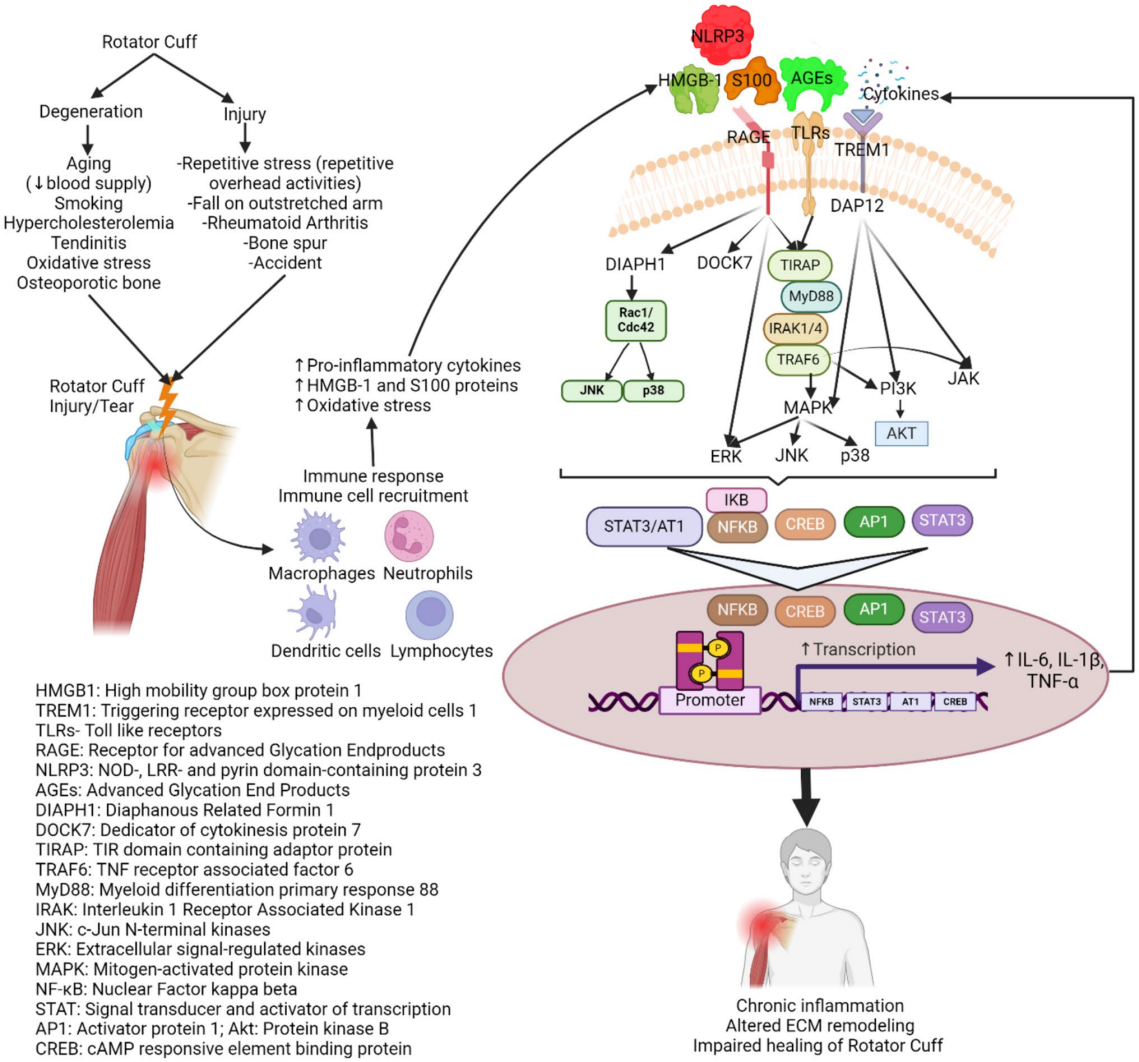
Pathophysiology of rotator-cuff tear and the inflammatory signaling involving RAGE and TLRs. The immune response of the body to rotator-cuff tear either due to degeneration or injury causes increased infiltration of immune cells followed by increased secretion of DAMPs including HMGB-1 and S100 proteins. These DAMPs and other ligands of TLRs, TREM-1, and RAGE including cytokines and AGEs activate downstream signaling promoting increased secretion of pro-inflammatory cytokines perpetuating a cascade of inflammation contributing to chronic inflammation of the tendon and delayed tissue healing

Inflammation and tissue degeneration serve as critical players in the progression of RCI. In response to injury or chronic overuse, inflammatory response leading to the recruitment of inflammatory cells and release of pro-inflammatory cytokines contribute to the breakdown of ECM components and compromise the overall structure and function of rotator-cuff tendons [21]. The intricately interconnected molecular players, including the RAGE, toll-like receptors (TLRs), S100 proteins, and high mobility group box protein-1 (HMGB1), synergistically intensify the inflammatory response, exacerbating tissue damage [8, 21] (Fig. 1). This results in altered cellular functions, increased oxidative stress, and altered matrix remodeling ultimately leading to the nonhealing of rotator cuff.

RAGE activation happens because the injured tissues release endogenous ligands known as Damage-Associated Molecular Patterns (DAMPs) such as S100 proteins, AGEs, and HMGB1 [8] (Fig. 1). These DAMPs bind and activate RAGE and initiate downstream signaling pathways linked to inflammation such as Ras/MEK/ERK1/2 (MAPK signaling pathway), Stress-activated protein kinases (SAPK)/Jun amino-terminal kinases (JNK), mitogen-activated protein kinases (MAPK/p38), phosphatidylinositol 3-kinase (PI3K)/protein kinase B (AKT), and glycogen synthase kinase-3 beta (GSK-3β) to induce persistent inflammation and tissue damage [8]. Increased synthesis and release of interleukin (IL)-1, IL-6, and TNF-α via activating transcription factors such as NF-κB, STAT3, activator protein 1 (AP-1), and early growth response protein 1 (Egr-1) results in a vicious cycle of inflammation. The downstream effectors of RAGE signaling in different types of inflammatory and vascular cells are diaphanous related formin 1 (DIAPH1) [32], dedicator of cytokinesis (DOCK 7), extracellular signal-regulated kinase (ERK ½) [33], Toll/Interleukin-1 receptor domain-containing adaptor protein (TIRAP), etc. [34, 35]. The inflammatory response is mediated by cytokines, chemokines, and other mediators, which are produced by these pathways activating pro-inflammatory genes (Fig. 1). Due to their partial involvement in tissue repair, the exact function of RAGE activation in muscle injury is highly complex. RAGE activation may play a role in the differentiation and activation of satellite cells and other myogenic precursor cells into myoblasts, in which myoblasts facilitate the regeneration and repair of muscles [36]. After a muscle injury, RAGE activation aids in the recruitment of immune cells to the site of injury, including neutrophils and macrophages, promoting the inflammation required to remove cellular debris and start tissue-repair processes. However, in the presence of recurrent injury or comorbid conditions, these signaling cascades enhance the inflammatory response and lead to chronic inflammation [2, 8, 21, 37].

## RAGE: molecular understanding and significance

### Structural and molecular characteristics of RAGE

**Fig. 2:**
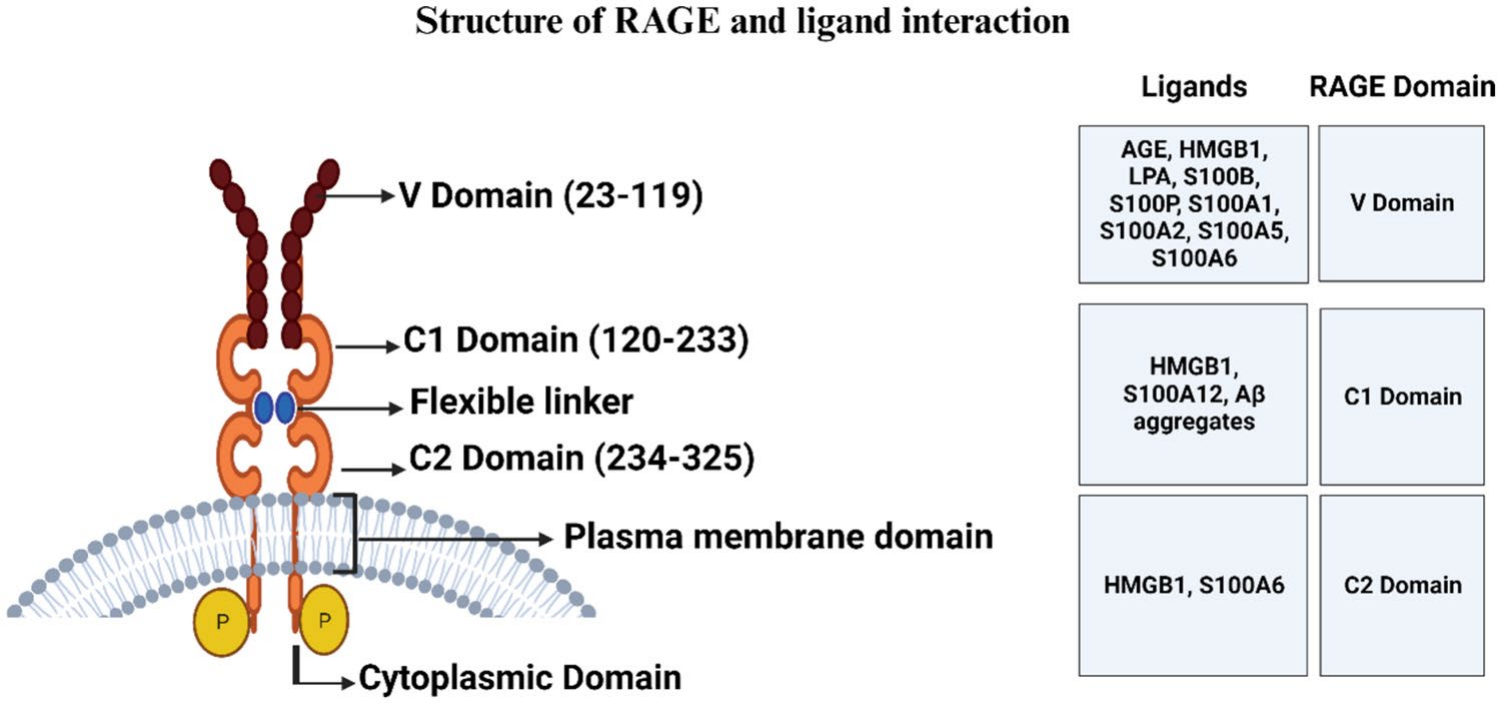
*Schematic representation of RAGE*. The extracellular portion consists of the V, C1, and C2 domains, which are immunoglobulin-like domains. The brief intracellular domain is joined to the extracellular domain by a single transmembrane helix. RAGE ligands engage in multiple pathways of interaction with the extracellular domains that mediate inflammation. AGEs: Advanced glycation end-products; HMGB1: High mobility group box-1 protein; LPA: Lysophosphatidic acid; Aβ aggregates: Amyloid Beta aggregates

A member of the Immunoglobulin superfamily, RAGE is a multi-ligand receptor whose structure is composed of three segments: the extracellular, transmembrane, and intracellular [18]. The extracellular segment of RAGE is the ligand binding site and is made up of three immunoglobulin domains: the V-type, C1-type, and C2-type domains (Fig. 2) [18]. RAGE’s interaction with various ligands including advanced glycation end products (AGEs), HMGB1, S100 proteins, and β-amyloid peptides, etc., activating different pathological inflammatory pathways is primarily facilitated by its VC1 domain. Furthermore, the self-association of the V − V or C1 − C1 domains may mediate a ligand-driven multimodal dimerization or oligomerization of RAGE. C2 is a separate structural unit that is flexibly linked to C1 by a linker that is 12 residues long. The C2 structure seems to have a substantial negatively charged surface as well as acidic residues pointing in the direction of the VC1 oligomer’s basic surface. Moreover, its cytoplasmic tail interacts with intracellular signaling molecules, enabling the transduction of signals within the cells [38]. The sequence identities of the extracellular domain (VC1C2) of human RAGE (UniProtKB Q15109) with mice (Q62151), rats (Q63495), and primates (Rhesus macaque; F1ABQ1) are 79.6%, 79.2%, and 96.5%, respectively.

## Signaling pathways depending on RAGE in different cell types

RAGE is expressed widely in various cell types including vascular endothelial cells, immune cells (VSMCs), monocytes/macrophages, neurons, cardiomyocytes, adipocytes, glomerular epithelial cells, podocytes, and alveolar epithelial cells. RAGE expression levels also vary in different cells in normal and pathological conditions like at low levels in a wide range of differentiated adult cells and high levels in mature lung type-I pneumocytes and skeletal muscle satellite cells in ischemic tissue [22, 39, 40]. The fact that RAGE signaling is rather complicated and that the cellular reaction to RAGE activation lacks an overall scheme is not surprising. Several distinct factors affect the result of RAGE signaling. In addition to the ligand’s identity, other factors that affect the cellular response that follows ligand engagement include the type of cell, ligand concentration, presence of other ligands, surface concentration of RAGE, potential co-receptors, various adaptor molecules that mediate the signal, and established signaling pathways within the cell.

### RAGE activation on endothelium

**Fig. 3:**
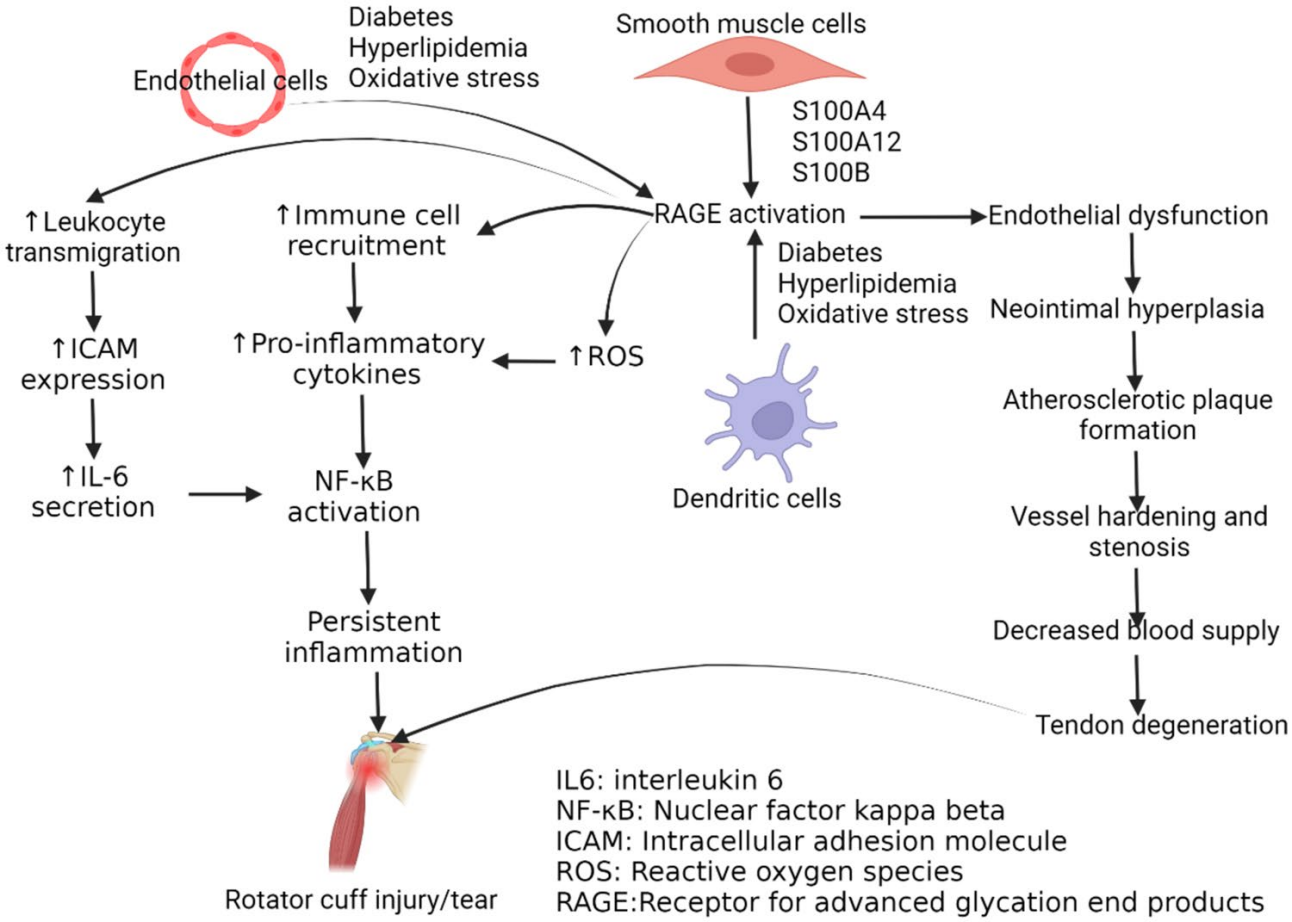
RAGE activation on endothelial cells, smooth muscle cells, and dendritic cells contributes to rotator cuff injury (RCI) or tendon tear. RAGE activation induces increased recruitment of immune cells, increased oxidative stress, and endothelial dysfunction causing increased persistent inflammation and decreased blood supply precipitating tendon degeneration. This hypothesis needs to be investigated involving tendon tissue after injury and repair both using animal models

RAGE plays a role in the recruitment of leukocytes, with the leukocyte adhesion molecule macrophage-1 antigen (Mac-1) controlling leukocyte recruitment during acute inflammation. In Alzheimer’s disease (AD) patients, T- cell infiltration is facilitated by RAGE recognition, promoting C–C motif chemokine receptor 5 (CCR5) expression on the blood brain barrier. Thus, RAGE activation on endothelium promotes leukocyte transmigration into inflamed tissues and endothelial dysfunction [41]. RAGE-induced leukocyte migration and vascular remodeling have been linked to diabetes-induced cognitive decompensation. Studies show increased expression of RAGE in neurons and glial cells in diabetic mice, leading to cognitive dysfunction and impaired hippocampal-dependent spatial memory, suggesting that RAGE expression is crucial for these processes. Leukocytes that exhibit RAGE signaling activate NF-κB, which in turn causes RAGE expression to persist and cells to become activated [22] (Fig. 3). Increased leukocyte migration to the area of inflammation after RAGE activation is mediated by increased expression of intracellular adhesion molecules (ICAMs) in the endothelial cells and via stimulation of Th2 cell differentiation leading to Th2 type inflammation by increased secretion of IL-6 [42]. Increased expression of ICAM is associated with cytokines-related inflammation and fibrosis in synoviocytes and adhesive capsulitis of the shoulder joint [43]. Adhesive capsulitis is a risk factor for rotator-cuff tears as well as a complication or rotator-cuff tear following surgical repair [44–46] (Fig. 3). Thus, the role of adhesive capsulitis and the role of RAGE-mediated ICAM activation should be investigated to design better therapeutics.

Activation of endothelial cells will increase the production of ROS-activating cellular signaling and contributing to inflammation via increased production of pro-inflammatory cytokines and B-cell activating factors through activation of transcription factor NF-κB involving MAPK pathway [20] (Fig. 3). For the formation of RAGE multimers, RAGE preassembly in the plasma membrane is essential. In disorders mediated by RAGE, elevated concentrations of RAGE on the surface stimulate hyperactivation, which leads to persistent inflammation. Inflammation plays a critical role in the pathogenesis and nonhealing of RCI, as discussed above.

RAGE activation of endothelial cells is associated with endothelial dysfunction and intimal hyperplasia obstructing the vessel and decreasing the blood supply to the organ [47] and treatment with sRAGE attenuates neointimal hyperplasia. Binding of RAGE with AGEs stimulates endothelial cells which produce ROS-activating NF-κB involving DIAPH1. Activation of NF-κB will contribute to arterial stiffness, endothelial dysfunction, and altered ECM remodeling compromising the blood supply [48] (Fig. 3). As shown in Fig. 1, decreased blood supply to tendon precipitates tendon degeneration, RAGE activation in endothelial cells in the vessels may contribute to it and sRAGE and other drugs targeting RAGE may be an option to investigate in RCI. RAGE activation on endothelial cells is associated with accelerated aberrant autophagy, promoting endothelial–mesenchymal–transition (EndMT) and contributing to tissue fibrosis. RAGE activation can also induce tissue fibrosis involving EndMT via activating TGF-β signaling, Notch pathway, Wnt pathway, and Smad3 [49]. As discussed above, fibrosis of the rotator-cuff tendon may increase load-wearing capacity but interferes with proper healing response. Therefore, the role of EndMT and involved signaling should be investigated to delineate a potential therapeutic target involving RAGE.

### RAGE activation on SMCs

Smooth muscle cells (SMCs) exhibit surface expression of RAGE and proliferate and migrate when stimulated by AGE or S100 proteins [50]. RAGE signaling on SMCs mediates atherosclerosis, post-vascular injury, and arterial pathologies in a diabetic milieu [51]. While S100A4 activates the MAPK pathway via RAGE, resulting in the expression of MMP2, which is crucial for vascular SMC migration by activating c-Src kinase and enhancing cell calcification, AGE-induced activation of RAGE initiates the MAPK pathway involving ERK1/2 in pulmonary SMCs [50]. Such atherosclerotic plaque calcification is linked to a reduction in mechanical stability and can cause acute cardiovascular dysfunction and plaque rupture. As discussed above, the AGEs-RAGE axis stimulates TGF-β which in turn can stimulate the proliferation and migration of VSMCs contributing to arterial hardening and decreased blood supply [52]. Vessel stenosis due to plaque formation will attenuate blood supply to the shoulder tendon causing degeneration and tear. RAGE activation on VSMCs and other cells including endothelium, monocytes, and T cells involving S100A12, S100B, and RAGE will contribute to increased immune-cell recruitment, secretion of pro-inflammatory cytokines, and inflammation [39] (Fig. 3). The findings of increased expression of alpha-smooth muscle actin and activation of SMCs in RCI suggest that SMC activation contributes to RCI by retraction of torn ends of the rotator cuff [53] (Fig. 3). These findings suggest that activation of SMCs may contribute to inflammation, arterial hardening, and retraction of tendon ends all contributing to RCI pathology and impaired healing.

### RAGE activation on monocytes

Monocytes are cells that are produced in the bone marrow and travel in the blood. They can quickly move into affected tissues and turn into macrophages during inflammation or injury. The activation of RAGE is dependent on the type of ligand and the differentiation state of monocytes/macrophages, indicating a highly regulated cellular response. Monocytes express RAGE, which plays a key role in their activation and differentiation. When activated by certain substances, such as S100B, monocytes release proinflammatory cytokines and chemokines [54]. HMGB1, released by injured tissues, stimulates monocyte invasion and chemotaxis to inflammatory sites [37]. In addition, HMGB1 triggers monocytes to produce and release more HMGB1, creating an autocrine/paracrine activation loop for transmigration [37]. The presence of RAGE on macrophages induces a proinflammatory response of macrophages via NF-κB activation, leading to M1 polarization characterized by high levels of major histocompatibility complex II (MHCII) and release of cytokines [55]. In diabetic mouse models, elevated levels of AGEs induce macrophage activation and a shift to the M1-polarized phenotype [56]. Several studies show that stimulation of cells with AGEs or HMGB1 induces signaling cascades, including protein kinase C (PKC [57], ERK1/2 [58], and MAPK p38 [18], leading to NF-κB activation. Activation of monocytes by AGEs under hypoxic conditions triggered membrane translocation of PKCβII, leading to phosphorylation of JNK and expression of transcription factor Egr-1 [59].

### RAGE activation on dendritic cells

RAGE plays a central molecular role in the migration and maturation of dendritic cells (DCs). DCs play a crucial role in the immune response by linking the innate and adaptive immune systems [60]. RAGE expressed on DCs is activated by ligands such as HMGB1, which promotes their maturation and migratory capacity. HMGB1 also induces the late activation of signaling pathways resulting in the activation of NF-κB pathway [61]. The sustained activation of MAPK and NF-κB is important for the migratory capacities of these antigen-presenting cells [62]. Increased expression and activation of DCs contribute to atherosclerotic plaque formation [63]. As stated above, plaque formation attenuates blood supply which contributes to rotator-cuff degeneration, the role of activation of DCs and related signaling in RCI should be investigated. This notion is supported by the fact that DCs are recruited to muscle-injury sites as an immune response [64] and an injury to the rotator-cuff muscles contributes to RCI and impaired healing (Fig. 3).

### RAGE activation on neuronal cells

Amyloid-β peptide (Aβ) is recognized by RAGE on neurons, microglia, astrocytes, and vessel wall cells. Alzheimer's disease (AD)-affected brain regions have higher levels of RAGE expression, and Aβ-RAGE interaction in vitro causes cell stress that activates downstream signaling pathways like the MAP kinase pathway and produces ROS [65]. Aβ induces long-term potentiation inhibition in entorhinal cortex slices through RAGE-mediated activation of p38 MAPK in neurons. Using a transgenic mouse model of AD-type, elevated expression of RAGE in an Aβ-rich environment accelerates and accentuates pathologic, biochemical, and behavioral abnormalities when compared to mice overexpressing only the mutant amyloid-β protein precursor, indicating its role in AD. The role of activation of RAGE on neuronal cells should be investigated because peripheral, spinal, and supraspinal neural factors play an important role in regulating tendon tear-related structural and functional muscle changes, effects on surgical repair and rehabilitation, impairment of shoulder function, and pain generation and management [66].

The activation of RAGE regulating migration and infiltration of immune cells including monocytes and DCs, which in turn are involved in RCI [25], suggests that targeting activated RAGE might be therapeutic. Further, neo-angiogenesis is very important for wound healing and regeneration of tissue [67]. The activated RAGE regulating proliferation and function of endothelial cells and VSMCs which are involved in angiogenesis [40] indicate that RAGE may play a critical role during healing of the injured tissue in RCI after surgery. It should be noted that, as discussed above, continued overexpression of RAGE is associated with persistent inflammation and atherosclerosis but activated RAGE is needed for the immune response regulated by monocytes and DCs. This suggests that timing of targeting RAGE after RCI is very important and targeting RAGE during the later phase of inflammation may be beneficial. Within immune cells, RAGE activation leads to oxidative stress and an increase in ROS [68]. RAGE signaling enhances the immune response by encouraging immune-cell migration and adhesion to inflammatory sites. This affects the health and repair of muscles by intensifying the inflammatory response. It may be possible to treat musculoskeletal disorders linked to immunological dysregulation and inflammation by focusing on RAGE-signaling pathways.

### Involvement of RAGE in the musculoskeletal system

The presence of RAGE in adult skeletal muscle is not typical, but it is briefly expressed in satellite cells when muscles are injured. RAGE expression on satellite cells is important for angiogenesis during regeneration [40]. RAGE signaling has various effects, including repressing the transcription of Pax7 in satellite cells and increasing the expression of myogenin (MyoG), a transcription factor that regulates myocyte fusion to promote myogenesis. This process speeds up muscle regeneration by facilitating myocyte fusion and restricts the self-renewal of satellite cells. In mice, lacking RAGE, satellite cells exhibit deficiencies in responding to stimuli and show delayed regeneration of injured muscles, underscoring the significance of RAGE in these processes [69].

Although protein levels drop significantly during the later stages of differentiation in vitro, RAGE mRNA expression remains consistent in proliferating and differentiating myoblasts. The fate of myoblasts seems to be influenced by the density of RAGE molecules on the cell surface and the relative concentration of HMGB1 and other RAGE ligands [70]. Muscle degeneration is associated with rotator-cuff tear and muscle regeneration and repair may help in tendon repair after RCI [71, 72]. This suggests that targeting MYOG and genes regulated by these transcription factors may be a potential therapeutic target to promote tendon repair. The signaling pathways related to MYOG in RCI warrant further investigation.

## RAGE is essential for tissue regeneration in the early stages of injury

**Fig. 4:**
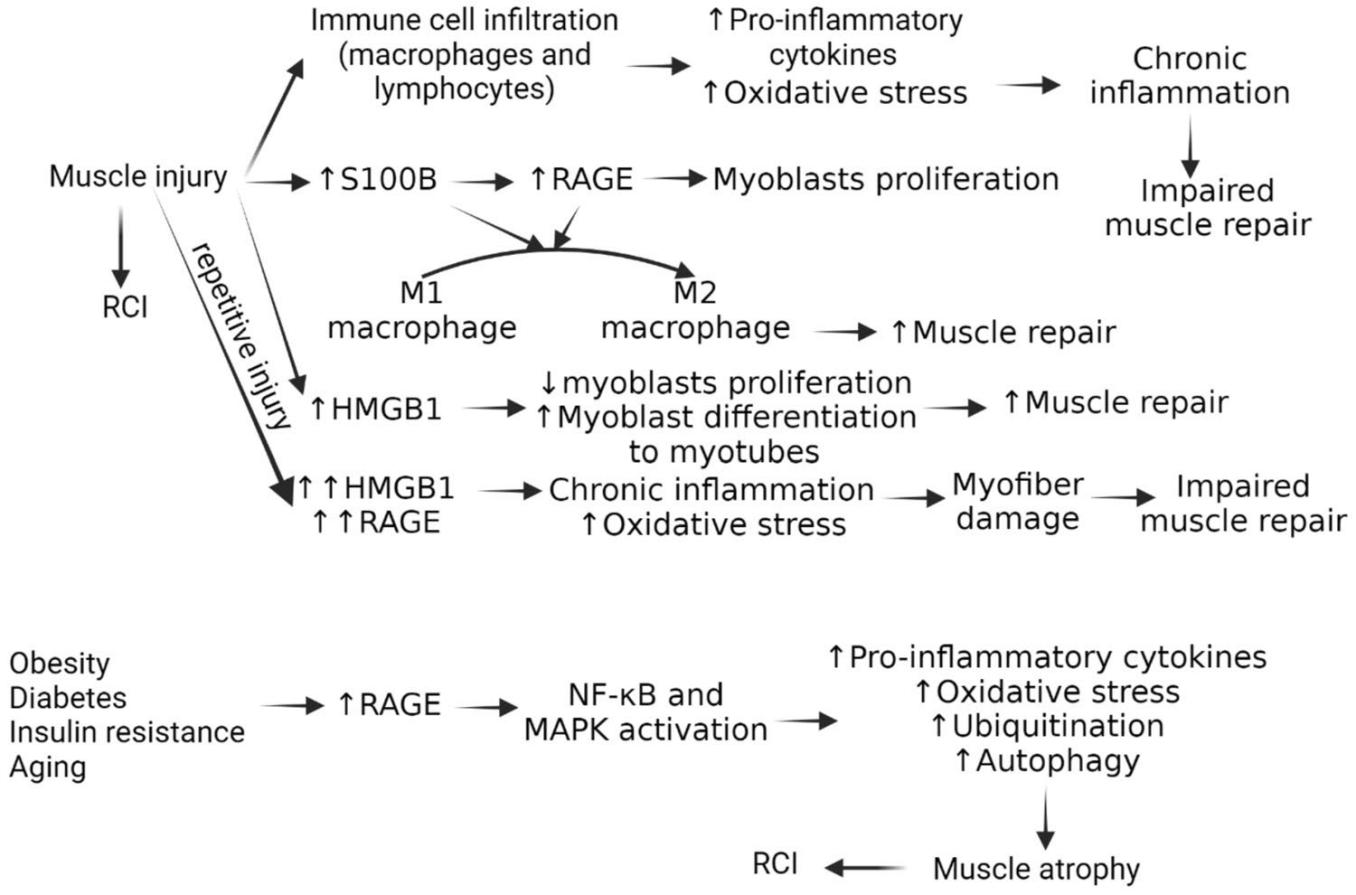
Schematic diagram showing the role of muscle injury and muscle atrophy in the pathogenesis of rotator-cuff injury. Receptor for advanced glycation end-products (RAGE), toll-like receptors (TLRs), S100 protein B (S100B), high mobility group box protein-1 (HMGB1), advanced glycation end products (AGEs), nuclear factor- kappa beta (NF-κB), rotator-cuff injury (RCI), and mitogen activated protein kinase (MAPK)

Early release of S100B from injured skeletal muscles stimulates RAGE signaling and increases RAGE expression in activated satellite cells, the quiescent adult stem cells (SCs), resulting in the proliferation of myoblasts and the activation of the myogenic program [73]. Moreover, the S100B/RAGE axis stimulates macrophage polarization to the M2 phenotype and macrophage infiltration in the acutely injured skeletal muscle. In the early stage after muscle tissue is damaged, HMGB1 is passively released and gradually accumulates during the regeneration process [36]. When HMGB1 activates RAGE, it stops proliferation and encourages myoblasts to differentiate into myocytes that can either form new myofibers or repair damaged ones (Fig. 4). Muscle injury triggers the release of HMGB1 from injured cells or immune cells, which interacts with RAGE on muscle cells or immune cells to initiate repair. This binding activates various signaling pathways, stimulating muscle stem cell proliferation and differentiation. The HMGB1-RAGE interaction also resolves inflammation, promoting tissue remodeling and regeneration. However, excessive inflammation can hinder healing. RAGE-mediated signaling in macrophages entering injured muscles speeds up the healing process, while sustained activation of RAGE by HMGB1 can worsen tissue damage and impair healing in chronic conditions [18].

RAGE ligands accumulate in the serum and damage myofibers during muscle diseases marked by high glucose levels, oxidative stress, or chronic inflammation [36]. Hence overstimulated and overexpressed RAGE causes lethal accumulation of oxidative stress that contributes to chronic myofiber damage, induces apoptosis in myocytes, and amplifies the inflammatory response by stimulating the secretion of proinflammatory cytokines [36]. Consequently, RAGE activation by HMGB1 in myoblasts not only promotes differentiation but also causes cell-proliferation arrest via p38 MAPK-mediated inhibition of the Raf-MEK-ERK1/2 pathway (Fig. 4). This results in the downregulation of cyclin D1 expression, phosphorylation of Rb (retinoblastoma suppressor protein), upregulation of the proliferation inhibitor p21WAF1, and inactivation of c-Jun N-terminal kinase (JNK). Furthermore, the anti-apoptotic factor Bcl-2 (B-cell lymphoma 2) expression is decreased in myoblasts through the HMGB1–RAGE interaction, which induces apoptosis [74]. Thus RAGE influences muscle regeneration in acutely injured muscles through two different mechanisms: it stimulates macrophage infiltration and polarization to the M2 phenotype and controls SC/myoblast activity, which permits appropriate myoblast proliferation and differentiation phases [36] (Fig. 4). However, excessive RAGE signaling brought on by S100B accumulation (as in muscular dystrophy) causes an overabundance of macrophage infiltration and an extension of the inflammatory phase, which ultimately causes disrupted regeneration [75].

### RAGE and skeletal muscle atrophy

A substantial loss in muscle mass, myofiber area, protein content, regenerative ability, and muscle strength, along with an increase in myocyte apoptosis, are the hallmarks of skeletal muscle wasting, or atrophy [76]. This process is intricate and tightly controlled. It typically coexists with other variables like aging, diabetes [77], denervation, and a variety of chronic diseases marked by various levels of systemic chronic inflammation, wherein morbidity and mortality are worsened by muscle atrophy. Skeletal muscles in young, healthy people retain mass and function using complex cellular networks that are in balance and control the synthesis and breakdown of muscle proteins (hypertrophy and atrophy), resulting in physiologic levels of muscle proteins. This balance changes to a catabolic state during muscle atrophy, which causes the breakdown of myofibrillary proteins. Discussing muscle atrophy is important because it is associated with RCI and the degree of supraspinatous muscle atrophy depends on the size of the tear, aging, and disuse [78, 79]. A study using rat model suggest that treatment of muscle atrophy after RCI using electroconductive nanofibrous matrices may be beneficial [80].

RAGE in insulin resistance has been connected to muscle atrophy. Insulin resistance has been linked to RAGE activation, which may have an indirect effect on muscle health [81]. Insulin resistance can impair muscle growth and maintenance by interfering with protein synthesis. When RAGE is activated by its ligands, such as AGEs, it can start pro-inflammatory signaling pathways like NF-κB that contribute to the synthesis of inflammatory mediators. RAGE signaling has been linked to the control of several biologic processes, including the activation of mechanisms like the ubiquitin–proteasome system and autophagy that break down muscle proteins. Muscle wasting may result from overactivation of these pathways. Increased protein degradation and impaired muscle function are linked to elevated oxidative stress in muscles [82] and this can lead to muscle atrophy by inducing p38 MAPK/myogenin axis activation and STAT3-dependent degradation of MyoD1 (myoblast determination protein 1) in various disorders such as muscular dystrophies, aging and sarcopenia as well as disuse atrophy [83]. (Fig. 4) The musculoskeletal system can be impacted by insulin resistance and metabolic dysfunction, which can alter the strength and integrity of muscles, tendons, and other connective tissues [84]. Weakened and deteriorated tissue may make a subject more vulnerable to ailments like rotator-cuff tears. Thus, targeting insulin resistance may be an avenue to improve muscles in the shoulder joint which ultimately will promote repair in RCI.

Obesity is a common comorbidity among those who have insulin resistance [85]. Obesity can lead to poor posture, changed movement patterns, and increased joint stress, which can put an individual at risk for injuries like rotator-cuff problems because it changes their biomechanics and puts more strain on their shoulder. Insulin resistance in obesity leads to hypertrophied adipose tissue, causing fat cells to increase in size and release proinflammatory cytokines [86]. This excess inflammation contributes to chronic low-grade inflammation, which is crucial for insulin resistance, type-2 diabetes progression [87], and complications like cardiovascular diseases, neuropathy, nephropathy, and impaired wound healing [88] (Fig. 4).

Chronic low-grade inflammation and elevated RAGE ligand accumulation are associated with age-related muscle atrophy and sarcopenia [36, 89, 90]. Feeding young mice with a diet high AGEs was found to impair their skeletal muscle growth and contractile function [91]. The promotion of RAGE-mediated 5’-adenosine monophosphate-activated protein kinase (AMPK) down-regulation by AGEs results in skeletal muscle atrophy. RAGE inhibition has positive effects on several kinds of aging-related pathologies, such as cancer [92], diabetes [93], cardiovascular disease [94], and neurodegeneration [95]. Loss of muscle and bone can be prevented by blocking RAGE signaling. Blocking cellular RAGE using soluble RAGE consistently prevented alveolar bone loss in diabetic periodontal disease-affected mice. Muscle-satellite cells isolated from elderly individuals also showed high intracellular concentrations of RAGE ligand S100B, which is known to inhibit myogenic differentiation by repressing the myogenic regulatory factors MyoD and myogenin [73]. This points to a possible role for RAGE signaling during the development and progression of aging-related bone and muscle pathology, as well as a therapeutic opportunity to target and inhibit this signaling.

Inflammatory myopathies, facioscapulohumeral muscular dystrophy, and limb-girdle muscular dystrophy (LGMD) all indicate re-expression of RAGE in the muscles. The muscles of mdx mice, an experimental model of Duchenne muscular dystrophy (DMD), express elevated RAGE expression [96]. These mice also release large amounts of HMGB1 and S100B, two RAGE ligands. A double mutant, mdx/Ager^–/–^ mice devoid of RAGE and dystrophin, exhibit reduced inflammation, intact fibrosis, and increased muscular strength. Mdx/Ager^–/–^ macrophages exhibit reduced reactivity to proinflammatory stimuli and exhibit reduced expression of C–C chemokine receptor 2 (CCR2), C–C chemokine ligand 2 (CCL2), and CCL7, all of which are crucial for the chemotaxis and migration of monocytes and macrophages. RAGE expression in activated immune cells, such as T and B lymphocytes and macrophages induces inflammatory cell infiltration, cytokine production, and NF-κB activation, all of which contribute to the acceleration of inflammation [97]. The interaction of RAGE activation and oxidative stress has a major effect on muscle protein turnover and function in several diseases, including sarcopenia associated with aging, disuse atrophy, and some muscular dystrophies[98] (Fig. 4). Treatment strategies targeted at preserving muscle mass and function may involve RAGE-induced dysregulation of signaling pathways in these underlying conditions. Treatments that modulate these pathways by targeting RAGE ligand interaction could potentially be able to reduce the muscle atrophy and dysfunction linked to elevated oxidative stress in these conditions.

### RAGE activation after muscle injury

Upon skeletal muscle injury, different inflammatory cells are activated. When there is cellular stress or injury, DAMPs are released into the extracellular environment and blood circulation and are recognized by pattern recognition receptors (PRRs) like Toll-like receptors (TLRs) and scavenger receptors (SRs) or non-PRRs like RAGE [99] that are expressed on immune cells. When leukocytes recognize DAMPs, downstream signaling is activated through the activation of transcription factors nuclear factor κB (NFκB) and activator protein 1 (AP-1) and enhances the production of inflammatory cytokines [100] (Fig. 1). When a RAGE blocking antibody is used to treat dystrophic muscles in vivo, there was less necrosis and inflammatory infiltrates.

This suggests that the modulation of RAGE activity may be therapeutically important for skeletal muscle in a variety of diseases where oxidative stress and chronic inflammation are common denominators (Fig. 4). Further, the possibilities of targeting muscle injury and muscle atrophy and the underlying molecular factors should also be investigated to design better therapeutics improving clinical outcomes.

## RAGE activation in the rotator cuff: impact on cellular responses and tissue homeostasis

**Fig. 5:**
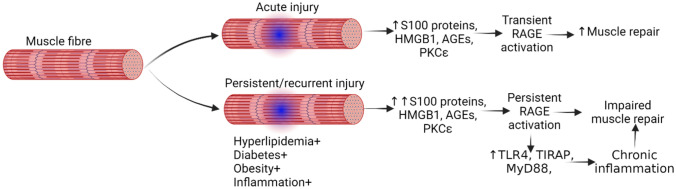
RAGE in the pathogenesis of muscle injury. Transient RAGE activation after muscle injury may be beneficial in promoting repair via acute inflammatory response while chronicity will lead to impaired muscle repair causing decreased tendon strength. Thus, targeting factors involved in muscle repair may therapeutically be beneficial in promoting tendon health as exercise improves tendon health. RAGE, toll-like receptor 4 (TLR4), high mobility group box protein-1 (HMGB1), advanced glycation end products (AGEs), toll/Interleukin-1 receptor domain-containing adaptor protein (TIRAP), myeloid differentiation primary response protein 88 (MyD88), and protein kinase C epsilon (PKCε)

RAGE has been studied in several musculoskeletal disorders, such as sarcopenia, atrophy, dystrophy, and muscle injury (Fig. 4). Muscle atrophy is linked to increased muscle protein breakdown and compromised muscle function following muscle damage, which is caused by uncontrollably activating RAGE through the release of DAMPs [36]. As shown in Fig. 4 that immune response via increased RAGE activation is beneficial for muscle regeneration, however, persistent activation or overstimulation of RAGE is detrimental to muscle repair. This suggests that depending on the type and duration of injury, the pathogenesis of response varies, and each has a different outcome (Fig. 5). Acute inflammatory response promotes muscle regeneration while persistently increased RAGE expression after muscle injury or muscle atrophy causes oxidative stress and inflammation, which further damages muscle fibers [101, 102]. The presence of comorbid conditions further adds to this continuum and delay in the tendon repair aggravates RCI [103, 104].

As discussed above, AGE-RAGE interaction is a critical factor in the pathogenesis of rotator-cuff injuries and other tendon disorders. Localized accumulation of Advanced Glycation End products (AGEs) and the activation of their receptor, RAGE, are significantly elevated in tendinopathic tissues [105]. This interaction likely contributes to intrinsic risk factors for tendinopathy by altering tendon mechanics through enhanced COL crosslinking and reduced COL-turnover rates (altered ECM remodeling), ultimately decreasing tendon resilience to mechanical load and increasing the risk of injury.

A recent study on frozen shoulder (FS) using bioinformatics analysis revealed increased expression of ADAMTS1, NR4A2, PARD6G, and SMKR1 in FS onset and progression. These genes are involved in key biologic processes such as ECM organization, COL metabolism, and immune-cell infiltration: the processes relevant to RCI repair [106]. ADAMTS1 and NR4A2 are central to fibrosis and inflammation, which are crucial in both FS and RCI. Targeting these pathways and genes presents a promising therapeutic strategy for managing rotator-cuff injuries, particularly where inflammation and fibrosis are prominent. The insights into immune-cell infiltration and gene-expression profiles from this study provide a strong foundation for further exploration of novel biomarkers and treatment approaches for RCI, drawing parallels from FS pathology. Since ADAMTS1 promotes COL-fiber assembly/stabilization and COL release [107], targeting it in RCI may have therapeutic benefits because ECM remodeling is important for RCI repair [108].

A potential association between AGEs and tendon-stump classification in rotator-cuff injuries was recently investigated [24]. The study found that stump type 3 exhibited higher AGE accumulation, increased oxidative stress, apoptosis, and reduced cell viability, indicating greater rotator-cuff fragility. Stump type 3 also showed elevated levels of AGE, increased expression of RAGE and NOX, leading to higher oxidative stress and apoptosis. Histological analysis confirmed disordered COL orientation and increased type 3/1 COL ratio in type-3 stumps, which align with imaging assessments revealing higher intensity of edema, degeneration, and inflammation in type-3 stumps. These results again suggest that targeting AGE-RAGE axis and the factors involved may be therapeutically beneficial.

### Protein kinase C epsilon (PKCε) and RAGE signaling after muscle injury

Apart from these, additional factors secreted after muscle injury could potentially impact RAGE signaling. Protein kinase C epsilon (PKCε), a member of the Protein kinase C family, is one of the important signaling pathways contributing to RAGE activation [109] and signaling pathways and cellular processes, including inflammation, tissue repair, and injury. PKCε can regulate RAGE activation directly or indirectly. It could affect RAGE’s expression or activity, which could influence how effectively it binds to its ligands and regulates downstream signaling. PKCε could also phosphorylate the factors involved in RAGE-signaling pathway. The signaling cascades that are triggered by RAGE activation can be improved or altered by this phosphorylation [110].

PKCε activity might have an impact on the affinity of the ligands or availability for binding to RAGE, which would modify the signaling events that follow. PKCε phosphorylates the cytosolic domain of RAGE on Ser391 upon RAGE ligand binding, mediating interaction with the TIR domain TIRAP [111] (Fig. 5). TIR Domain Containing Adaptor Protein (TIRAP) serves as a link to MyD88 (myeloid differentiation factor 88), just like TLR signaling does (Fig. 1). RAGE activation causes proinflammatory signaling that is dependent on MyD88 in this manner. Later mutations in the TIR domain of TIRAP inhibit downstream inflammatory signaling induced by RAGE. Several ligands, such as HMGB1, S100 proteins, and others, activate TLR4 and RAGE simultaneously, triggering the same inflammatory pathway through the TIRAP interaction. It has also been shown that there is direct communication between the two signaling pathways [112, 113]. This suggests that targeting TIRAP may attenuate the effect of activated RAGE and TLR4 and whether the therapeutic intervention of TIRAP and RAGE represents a way to effectively treat these diseased states should be thoroughly investigated in the context of RCI.

The findings discussed above suggest that PKCε can affect cellular responses like oxidative stress, apoptosis, inflammation, and tissue repair by influencing RAGE-mediated signaling. However, the precise molecular mechanisms and degree of interaction between PKCε and RAGE in various type of cells, the context of injury or disease, and the ligands involved are not well understood. Understanding the correlation between RAGE and PKCε in the context of RCI and designing targeted therapies may be beneficial in improving the repair by attenuating inflammation and promoting muscle tissue repair. The notion of targeting PKCε to promote muscle repair is supported by the fact that PKCε is secreted after muscle injury and is a promoter of skeletal muscle differentiation and regeneration [114]. However, how improving muscle health and repair in RCI patients will improve RCI repair must be investigated.

## Clinical implications of RAGE in RCI and future directions

Rotator cuff pathology needs approximately 75,000 arthroscopic repair surgeries annually. The surgery can be beneficial for repairing severe tears or chronic conditions, but it has potential drawbacks like stiffness, reduced range of motion, persistent pain, and the risk of re-tearing or failing to heal. Recovery can be lengthy, requiring months of rehabilitation and physical therapy, which can be challenging for some individuals. Tendon to bone healing after surgery is influenced by factors like hypercholesterolemia, age, smoking, diabetes, osteoporosis, and muscle atrophy. Targeting hyperlipidemia and optimizing rehabilitation with COL and biologic augmentation can improve surgical outcomes [2]. RCI symptoms can be managed through rest, activity modification, ice application, and physiotherapy. Lowering hyperlipidemia is crucial for repair after surgery. Common non-surgical treatment strategies include using statins, peroxiredoxin 5, and vitamin D. Statins can reduce inflammation and fibrosis, enhance tendon healing, and reduce tear risk [2]. However, they can also cause myalgia, muscle injury, and tendon ruptures [2, 115]. It may be possible to mitigate these problems and enhance clinical outcomes by targeting the novel mediators including TLR-4 signaling, TIRAP, RAGE signaling, and mediators involved in muscle injury, as discussed above.

To improve the clinical outcome, a study focused on identifying anterior inferior glenoid rim (GLAD) lesions in patients undergoing shoulder surgery [116]. The study reported that the “chicken-wing muscle up” test, designed to replicate the injury mechanism, demonstrated high accuracy in identifying GLAD lesions. However, the test produced false positives in cases of Bankart and SLAP injuries due to similar pain responses and movements. Despite this limitation, the study suggests that the “chicken-wing muscle up” test shows promise as a supplementary diagnostic tool, particularly for high-risk athletes. Further research is needed to validate and refine its use.

## Conclusion

An increased expression of AGEs and RAGE in RCI tendons and the involvement of RAGE in inflammation, tissue repair, and degeneration underscores its importance in the development and progression of RCI. Thus, targeting RAGE and its downstream signaling may offer novel approaches for the development of advanced therapeutic interventions for rotator-cuff injuries. An important aspect to promote tendon health is to target muscle health [117, 118] and targeting factors involved in muscle regeneration after an injury as adjuvant therapy, as discussed in this review including RAGE, TIRAP, MYOD1, PKCε, and DIAPH1, will be beneficial in promoting RCI health.

## Data Availability

The information presented in this article was collected from published reports in the literature. These references have been cited. No new data are generated. For any other information can be obtained from the corresponding author on reasonable request.
